# Exploiting the Features of Clinical Judgment to Improve Assessment of Disease Severity in the Emergency Department: An Acutelines Study

**DOI:** 10.3390/jcm13051359

**Published:** 2024-02-27

**Authors:** Martje Visser, Daniel Rossi, Hjalmar R. Bouma, Jan C. ter Maaten

**Affiliations:** 1 Department of Internal Medicine, University Medical Center Groningen, University of Groningen, 9713 GZ Groningen, The Netherlands; m.visser02@umcg.nl (M.V.); .; j.c.ter.maaten@umcg.nl (J.C.t.M.); 2 Department of Clinical Pharmacy and Pharmacology, University Medical Center Groningen, University of Groningen, 9713 GZ Groningen, The Netherlands; 3 Department of Aute Care, University Medical Center Groningen, University of Groningen, 9713 GZ Groningen, The Netherlands

**Keywords:** clinical impression score, clinical judgment, clinical assessment, emergency department, risk management, sepsis

## Abstract

Background: Clinical judgment, also known as gestalt or gut feeling, can predict deterioration and can be easily and rapidly obtained. To date, it is unknown what clinical judgement precisely entails. The aim of this study was to elucidate which features define the clinical impression of health care professionals in the ED. Method: A nominal group technique (NGT) was used to develop a consensus-based instrument to measure the clinical impression score (CIS, scale 1–10) and to identify features associated with either a more severe or less severe estimated disease severity. This single-center observational cohort study included 517 medical patients visiting the ED. The instrument was prospectively validated.. The predictive value of each feature for the clinical impression was assessed using multivariate linear regression analyses to adjust for potential confounders and validated in the infection group. Results: The CIS at the ED was associated with ICU admission (OR 1.67 [1.37–2.03], *p* < 0.001), in-hospital mortality (OR 2.25 [1.33–3.81], *p* < 0.001), and 28-day mortality (OR 1.33 [1.07–1.65], <0.001). Dry mucous membranes, eye glance, red flags during physical examination, results of arterial blood gas analysis, heart and respiratory rate, oxygen modality, triage urgency, and increased age were associated with a higher estimated disease severity (CIS). On the other hand, behavior of family, self-estimation of the patient, systolic blood pressure, and Glascow Coma Scale were associated with a lower estimated disease severity (CIS). Conclusion: We identified several features that were associated with the clinical impression of health care professionals in the ED. Translating the subjective features and objective measurements into quantifiable parameters may aid the development of a novel triage tool to identify patients at risk of deterioration in the ED.

## 1. Background

Rating disease severity is crucial in the emergency department (ED), since the first clinical judgement can predict mortality and prognosis [1,2,3,4,5]. Early recognition of disease severity is essential for timely treatment and prevention of clinical deterioration, as demonstrated in sepsis [6,7,8,9]. Recognition of disease severity in patients with an undifferentiated complaint, such as early sepsis, and predicting the clinical course is difficult because signs and symptoms can be nonspecific and highly variable [8]. Experienced clinicians can recognize and correctly interpret the patient’s general appearance within a few seconds of interacting with an acutely ill patient [10]. However, risk stratification of patients with acute infection and sepsis remains a challenge for clinicians and nurses [11,12].

Besides the Emergency Severity Index (ESI) triage, several clinical scoring systems have been developed to provide early warning in acute illnesses, such as SIRS, qSOFA, NEWS, and, recently, NEWS2 [13,14,15,16]. These scores have limitations in terms of sensitivity and specificity in clinical utility in predicting clinical outcomes [4,17,18]. The clinically proposed cut-off of two or more of the qSOFA has a sensitivity of only 47% (95% CI, 0.28–0.66) and a specificity of 93% (95% CI, 0.88–0.97), respectively, for predicting sepsis in hospitalized patients [17]. Despite the availability of numerous tools to aid in the classification or diagnosis (acute) of diseases, clinical judgment remains the basis of clinical decision making and, for most tools, it remains unknown how these perform compared to clinical judgment [19]. Hence, there is a large unmet medical need for methods to rapidly improve the stratification of patients according to disease severity, identify its cause and stage, and stratify patients for targeted personalized treatment [19]. Clinical impression, also known as gestalt or gut feeling, is easily obtained of disease severity that has been shown in previous studies to be associated with future deterioration [4,14]. Nurses’ subjective feeling of concern is known to detect subtle changes in a patient’s condition even before vital signs have changed [20]. Most general practitioners (GPs) use their gut feeling in rating disease severity [21]. The clinical impression score, a single-question 10-point Likert scale, could be a promising tool to assess disease severity in the ED. This tool can be a fast and reliable method to stratify between ICU and general ward admission [4,5,10,14].

It remains unknown what the clinical impression precisely entails. Yet, unraveling the clinical impression is necessary to explore factors that contribute to our clinical impression, which could aid in the development of more precise systems for stratifying patients in the ED. We hypothesize that such factors may include both objective measurements (e.g., age and vital signs) and subjective features (e.g., estimated biological age and interpretation of behavior). The aim of the current study was to elucidate the subjective features and objective measurements that determine the clinical impression of the health care professional at the ED. First, using a nominal group technique, a consensus-based scoring instrument was developed by a group of ED experts to measure subjective features that determine the clinical impression. Second, this instrument was prospectively validated in the ED. Thereby, the predictive value of each feature for the clinical impression was assessed in 517 ED patients using multivariate linear regression analyses to adjust for potential confounders and validated in the infection group.

## 2. Methods

### 2.1. Study Design

First (phase 1), a nominal group technique (NGT) [22] was used to create a consensus-based instrument to measure the clinical impression and to identify specific features that determine the clinical impression. Clinical impression was measured as a score: the clinical impression score (CIS) ranging from 1 to 10 (not ill to severely ill; Supplementary Table S1 and Supplementary Figure S1). Next (phase 2), the consensus-based instrument was prospectively validated in the ED of the University Medical Center of Groningen (UMCG), a tertiary care teaching hospital.

### 2.2. Developing the Clinical Impression Score Instrument (Phase 1)

- Step one: Recruitment of participant for NGT session

Participants for the NGT workshop were selected after an invitation was sent to acute and emergency physicians. Participants who responded positively to this invitation were included: five acute physicians (71% responded) and two emergency physicians (28% responded).

- Step two: Delphi technique with the group of experts

The purpose of the brainstorming session was to define the nature of clinical judgment, to conceptualize factors and attributes that are believed to constitute it when assessing patients with suspected or proven sepsis, and to reach consensus on the factors. The session consisted of a presentation introducing the topic “Sepsis and Clinical Judgment”, discussing current and past consensus on definitions of sepsis [1,15], tools developed for diagnostics such as NEWS, SIRS, SOFA, and qSOFA, recent evidence on clinical judgment [4,13], and its implications for further research. During the workshop, participants were asked to answer the following research question: “What factors define clinical judgment in the early evaluation of patients with suspected infection/sepsis?” In answering this question, participants were asked to relive a situation in which an exemplary patient with suspected sepsis would arrive at the ED and were encouraged to use all their senses to analyze which factors would give them a good or bad “gut feeling”.

- Step three: Consensus-based final selection of factors

Participants consecutively voted unanimously whether an item should be included as a factor defining clinical judgment in the assessment of patients with suspected infection or sepsis. Only items with a consensus rate of 100% (7/7) consensus rate were selected for further inclusion. The items listed in Supplementary Table S1 under “Factors 2nd round” all received a consensus rate of 100%.

- Step four: Development of the clinical impression score questionnaire

The final step was to incorporate factors into a questionnaire. Items selected after the workshop were used with the clinical impression score to create the questionnaire, which was constructed in Dutch and reviewed by three team members (MV, HRB, and JCtM) (Supplementary Figure S1).

### 2.3. Prospective Validation of the CIS (Phase 2)

#### Population and Data Collection

Data for this cohort study were obtained from the Acutelines data biobank [23]. Acutelines is a multidisciplinary prospective hospital-based cohort study in the ED of the University Medical Centre Groningen (UMCG), a tertiary care teaching hospital in the northern Netherlands. The cohort population is broadly representative of the population with acute medical conditions in the northern Netherlands. Primary screening of patients for eligibility on arrival at the ED is performed 24 h a day by the ED nurse together with a trained research team. Participants are asked to give written informed consent, with a proxy if necessary. One of the researchers asked either the physician or the nurse to complete the CIS instrument after primary assessment in the ED. Data were collected and managed using REDCap (Vanderbilt University, Nashville, TN, USA) electronic data capture tools hosted at the UMCG [24,25].

Bedside monitoring data were automatically captured and stored, and information from other data sources including the electronic health records of the hospital was securely imported via the electronic patient file (EPIC systems, Boston, MA, USA).

Patients for the current study were included from 20 September 2020 until 1 September 2021.

### 2.4. Ethical Approval

The full Acutelines’ protocol and an overview of the current, full data dictionary are available at www.acutelines.nl (accessed on 1 September 2020). Acutelines has been approved by the UMCG Medical Ethics Committee and is registered on ClinicalTrials.gov. under trial registration number NCT04615065. This study is conducted in accordance with the Declaration of Helsinki and is registered under research/registry number 202100635 and date of approval 13 September 2021.

### 2.5. Inclusion and Exclusion Criteria

Patients were eligible if they arrived for the specializations internal medicine, nephrology, geriatric medicine, oncology, hematology, pulmonology, rheumatology, gastrointestinal/liver medicine, urology, or emergency medicine (non-trauma). Adult patients (≥18 years) from these specializations were screened. Patients were excluded from participation if they were transferred to another hospital after initiation of treatment, were immediately discharged from the ED, visited the ED due to accidental contact with patient material, or visited the ED as a recipient of an organ transplant.

### 2.6. Definitions and Outcomes

The qSOFA and NEWS were calculated using the initial vital signs measured during admission to the ED. We retrieved data on mortality by checking the electronic hospital database. The primary outcome was the CIS (scale 1–10) (Supplementary Figure S1), whereas variables of the questionnaire were considered as predictors. Secondary outcomes were admission to hospital, ICU admission, in hospital mortality (<48 h), and 28-day mortality.

### 2.7. Statistical Analysis

Manual coding of the round-robin discussion transcriptions and writing “in silence” data was deemed unnecessary, as they were recorded and displayed in real time during the workshop itself. Data are presented as percentages, means, and standard deviation for normally distributed variables or as median with interquartile range for non-normally distributed variables. The Shapiro–Wilk test was used to test for normality. For binary data, χ^2^ was performed. For categorical data, the nonparametric one-sample test was performed using the binominal test or the Kolmogorov–Smirnov test.

Univariate logistic regression was performed to analyze the association of the CIS with different endpoints: hospital admission, ICU admission, in-hospital mortality (<48 h), and 28-day mortality. Subsequently, variables with a univariate association of *p* < 0.20 were selected for subsequent stepwise multivariate linear regression; variables with a *p*-value > 0.05 were excluded from the model. Statistical analysis was performed with IBM SPSS Statistics for Windows, version 28.0.

## 3. Results

### 3.1. Development of the Clinical Impression Score Instrument

Items selected at the NGT workshop were used with the clinical impression score (CIS) to create a questionnaire (Supplementary Table S1). The CIS is a straightforward score from 1 to 10: 1 indicates that the patient is not ill and 10 indicates that the patient is extremely ill. After completing the CIS, the health care professional was asked “Which items contributed significantly to your clinical impression?” The case record form was employed to obtain the clinical impression score and identify features associated with the clinical impression by physicians and nurses at the ED, consisting of 46 factors that might contribute to the clinical impression according to the expert panel (Supplementary Figure S1). The questionnaire was constructed in Dutch, which was the language fluently spoken by all ED staff. A translated version is shown in Supplementary Figure S1.

### 3.2. Prospective Validation of the Clinical Impression Score Instrument

Demographic data are shown in Table 1. A total of 517 patients met the inclusion criteria, of whom 307 (59%) patients presented with infection or sepsis, 55 (11%) with respiratory problems, or 26 (5%) with intoxication. A total of 355 (69%) patients arrived by ambulance, 258 (50%) had an Emergency Severity Index (ESI) triage color of orange, and 327 (63%) of the patients had an NEWS of three or more. In total, 399 (77%) patients were hospitalized, of which 46 (9%) were admitted to the ICU; 27 (5%) patients died within 28 days, of which 8 (1.5%) patients died within <48 h after hospital admission. The clinical experience of the health care professionals in this study ranged from 0 to 46 years. The median (IQR) years of work experience of the health care professionals was 6 (6) years. A total of 242 (48.5%) of the physicians were residents in their final years of training to become a medical specialist, with a median (IQR) of 5 (2) years of work experience. A total of 52 (10.4%) were specialists, with a median (IQR) of 11 (3) years of work experience. A total of 188 (37.7%) were nurses, with a median (IQR) of 12 (14) years of work experience. A total of 35 (6.8%) were medical interns. Role and years of experience were missing for 18 (3.5%) patients.

A logistic regression analysis was performed to assess the association between the CIS provided by the health care professional in the ED and clinical outcomes defined as hospital admission, ICU admission, in-hospital mortality (<48 h), and 28-day mortality. We demonstrated that the CIS was associated with ICU admission (OR 1.67 [1.37–2.03], *p* < 0.001), acute mortality (<48 h) (OR 2.25 [1.33–3.81], *p* < 0.001), and 28-day mortality (OR 1.33 [1.07–1.65] *p* < 0.001) but not with hospital admission (Table 2).

Next, to identify features associated with the CIS, the association between the features as identified by the NGT and the CIS provided by the ED health care professional was calculated (Supplementary Table S2). We then used a cut-off of *p* < 0.20 to select factors for subsequent stepwise multivariate linear regression analysis (Supplementary Table S3). Using this approach, we identified dry mucous membranes (OR 0.89 [0.47–1.35], *p* < 0.001), eye glance (OR 0.69 [0.32–1.06], *p* < 0.001), red flags on physical examination (OR 0.99 [0.62–1.35], *p* < 0.001), and arterial blood analysis (OR 0.53 [0.17–0.89], p 0.004) as independently associated with a higher CIS. On the other hand, behavior of the family (OR −0.66 [−1.13–−0.19], 0.006) and self-estimation of the patient (OR −0.59 [−0.97–−0.21], p0.002) were associated with a lower CIS. Together, these factors explained approximately 19% of variation in the CIS in the multivariate model (Table 3, R^2^ = 0.19 (adjusted = 0.19), df = 6, F = 19.09, *p* < 0.05). To adjust for age and disease severity, we adjusted the multivariate model using age, transport method, ESI triage urgency, and vital parameters, resulting in a model that explained approximately 40% of the variation in the CIS (Table 4, R^2^ = 0.42 (adjusted = 0.40), df = 13, F = 18.65, *p* < 0.05). Clinical impression features, vital parameters, and demographics associated with the clinical impression score in the multivariate regression model (*p* < 0.05, Table 4) were validated in 307 patients with infection by associating the factors with the clinical impression score in a multivariate linear regression analysis (enter). RC: regression score; CI: confidence interval. Model characteristics: R^2^ = 0.43, adjusted R^2^ = 0.40, df = 13, F = 14.82, *p* < 0.05 (Supplementary Tables S4 and S5).

## 4. Discussion

Here, we unraveled the majority of the factors that determine clinical judgment. First, a consensus-based instrument was developed, consisting of 46 factors that the expert panel thought to contribute to the clinical impression. Prospective validation of this instrument in medical ED patients revealed that dry mucous membranes, eye glance, red flags during physical examination, and interpretation of arterial blood gas results were independently associated with a higher estimated disease severity. On the other hand, behavior of family and self-estimation of the patient were associated with a lower estimated disease severity. When the model was adjusted for parameters indicative of disease severity, not only subjective factors but also objective measures such as ESI triage urgency, age, vital signs (i.e., heart rate, systolic blood pressure, respiratory rate, and blood oxygen saturation), and Glascow Coma Scale were independently associated with clinical impression. Together, we identified several subjective and objective factors associated with the clinical impression that explained almost half of the observed variability in the score.

Despite the presence of numerous tools to aid in the classification or diagnosis of (acute) diseases, clinical judgment remains the basis of clinical decision making and, for most tools, it remains unknown how they perform relative to clinical judgment [19,26]. Early recognition of disease severity is crucial for timely treatment and prevention of clinical deterioration, as demonstrated in sepsis [6]. However, despite the introduction of sepsis-3 (i.e., qSOFA) criteria in 2017 as a stratification tool in the ED, clinical judgment outperforms objective sepsis scores in predicting the clinical course of sepsis patients [4,13,14]. We have shown that the ED health care provider’s clinical impression is associated with the following clinical outcome: ICU admission and both in-hospital (<48 h) and 28-day mortality in patients with sepsis but also for patients with other acute conditions presenting to the ED. In addition, the clinical impression can be easily and rapidly obtained as compared to other scoring tools.

Discovering the components of clinical judgment may aid in the development of novel tools to improve the identification of patients at risk for deterioration in the ED. To our knowledge, no previous study has attempted to elucidate the factors associated with clinical impression. We identified nearly half of the variation (R^2^ = 0.40) in the clinical impression by including subjective factors and objective measurements in our model. When we compared the model with only subjective factors to the full model, we demonstrated a doubling of its performance, suggesting that at least one fifth of the clinical impression is determined by subjective factors and an equal amount is determined by objective measurements (i.e., age and vital signs). However, most of the variation in the clinical impression remains unknown. Previous studies have shown that intuition is one of the factors influencing clinical decision making [2,13,18]. Yet, “intuition” is difficult to quantify and may be at least partially captured by the subjective factors in our study [12,26,27]. In a recent study, it was shown that clinical judgement and any concerns about a patient’s condition should override the NEWS2 if the attending health care professional thinks that it is necessary to escalate [16].

In previous studies, we found moderate to good agreement in diagnostic accuracy of the CIS between physicians and nurses [4,13]. We do not know to what extent physicians and nurses take other factors into account in their clinical judgment. Together, while we were able to identify large differences in the clinical impression of physicians and nurses, the remaining unexplained factors governing the clinical impression may be due to intuition or personal characteristics (e.g., experience and skills).

## 5. Limitations

Potential limitations of our study include its single-center design in a tertiary care hospital with referral of patients for academic specialty care, which may limit its generalizability. Yet, our hospital has a substantial geographical spread in a rural area, ensuring a diverse population requiring both academic and nonacademic care. Furthermore, the use of a survey could potentially expose this study to both recall bias and reporting bias. First, varying levels of experience among the health care professionals may result in a wide range of clinical impression scores, thus introducing recall bias. Second, the time between the physician’s first clinical impression after the primary assessment and completion of the questionnaire could lead to reporting bias. However, to minimize this risk, the ED research team sought to obtain the clinical impression as soon as possible after primary assessment. In addition, all clinical impressions were provided while the patient was still in the ED.

## 6. Conclusions

We identified several features that influence the clinical impression of health care professionals in the ED. Translating the subjective features and objective measurements into quantifiable parameters may aid in the development of a novel triage tool to identify patients at risk of deterioration in the ED.

## Figures and Tables

**Table 1:** Demographics and distribution of covariates of the study population.

	Overall (N = 517)
Age (median (IQR))	69 (51.87)
Gender	
Male (n (%))	298 (58%)
Female (n (%))	219 (43%)
Charlson comorbidity index score (median (IQR))	4 (0.8)
No comorbidities (n (%))	55 (11%)
One or more comorbidities (n (%))	462 (89%)
Way of transport (N)	
Ambulance (n (%))	355 (69%)
Own transport (n (%))	158 (31%)
Helicopter (n (%))	1 (0.2%)
Already in hospital (n (%))	2 (0.4%)
Illness severity at ED presentation	
Emergency Severity Index triage category (median (IQR))	3 (2.4)
Red (n (%))	12 (2%)
Orange (n (%))	258 (50%)
Yellow (n (%))	247 (48%)
NEWS (median (IQR))	4 (0.4)
NEWS ≥ 3 (n (%))	324 (63%)
Infection group (N = 307)	307
qSOFA (median (IQR))	1 (0.2)
qSOFA 0 (low) (n (%))	148 (48%)
qSOFA 1 (moderate) (n (%))	126 (41%)
qSOFA ≥ 2 (high) (n (%))	33 (11%)
Diagnoses at the end of ED visit	
Infection or sepsis by physician (n (%))	307 (59%)
Respiratory problems (n (%))	55 (11%)
Cardiac problems (n (%))	20 (4%)
Gastro-intestinal problems (n (%))	30 (6%)
Syncope (n%)	22 (4%)
Intoxication (n (%))	26 (5%)
Electrolyte disbalance (n (%))	7 (1%)
Allergy (n (%))	8 (2%)
Other causes (n (%))	42 (8%)

IQR: interquartile range, NEWS: National Early Warning Score, qSOFA: quick Sequential Organ Failure Assessment.

**Table 2:** Association between CIS and clinical outcome.

Variabele	Overall (n (%))	OR (95% CI)	CIS *p*-Value
Number of patients	517 (100%)		
Clinical outcome			
Admission to hospital	399 (77%)	0.86 (0.52–1.43)	0.56
ICU admission	46 (9%)	1.67 (1.37–2.03)	<0.001
In-hospital mortality within 48 h	8 (1.5%)	2.25 (1.33–3.81)	<0.001
28-day mortality	27 (5%)	1.33 (1.07–1.65)	<0.001

Logistic regression analysis of the association of the CIS with different outcome measures. ESI = Emergency Severity Index triage system. Model characteristics of admission to hospital: X^2^ = 0.33, df = 1, −2 LL = 44.45, Rho = 0.01; *p* < 0.05. Model characteristics of ICU admission: X^2^ = 34.10, df = 1, −2 LL = 275.71, Rho = 0.14; *p* < 0.05. Model characteristics of in-hospital mortality within 48 h: X^2^ = 12.84, df = 1, −2 LL = 69.73, Rho = 0.17; *p* < 0.05. Model characteristics of 28-day mortality: X^2^ = 8.30, df = 1, −2 LL = 203.69, Rho = 0.05; *p* < 0.05. OR: Odds ratio, CI: confidence interval.

**Table 3:** Multivariate linear regression analysis of factors with clinical impression score.

Variables	Checked % (n/N)	RC (95%CI)	*p*-Value
Mucous membranes, dry	24.5% (126/515)	0.89 (0.47–1.35)	<0.001
Eye glance	63.6% (328/516)	0.69 (0.32–1.06)	<0.001
Behavior of the family	18.3% (93/507)	−0.66 (−1.13–−0.19)	0.006
Self-estimation of the patient	34.8% (179/514)	−0.59 (−0.97–−0.21)	0.002
Red flags at physical examination	57.3% (293/511)	0.99 (0.62–1.35)	<0.001
Interpretation of arterial blood gas analysis	52.3% (269/514)	0.53 (0.172–0.89)	0.004

Variables with a univariate association of *p* < 0.20 with clinical impression score were selected for stepwise multivariate linear regression analysis (Supplementary Table S2). Model characteristics: R^2^ = 0.19 (adjusted R^2^ = 0.19), df = 6, F = 19.09, *p* < 0.05. RC: regression coefficient, CI: confidence interval.

**Table 4:** Multivariate linear regression analysis.

Variables	Checked % (n/N)	RC (95%CI)	*p*-Value
CIS: Mucous membrane, dry	25% (126/515)	0.87 (0.42–1.132	<0.001
CIS: Eye glance	64% (328/516)	0.69 (0.30–1.108)	<0.001
CIS: Behavior of the family	18% (93/507)	−0.58 (−1.08–−0.08)	0.02
CIS: Self-estimation of the patient	35% (179/514)	−0.44 (−0.84–−0.03)	0.04
CIS: Red flags during physical examinations	57% (293/511)	0.99 (0.61–1.37)	<0.001
CIS: Interpretation of arterial blood gas analysis	52% (269/514)	0.44 (0.05–0.82)	0.03
Heart rate (n (%))	100% (517/517)	0.01 (0.002–0.02)	0.01
Systolic blood pressure (n (%))	517 (100%)	−0.01 (−0.02–−0.003)	0.01
Respiration rate (n (%))	416 (80%)	0.04 (0.01–0.07)	0.02
Oxygen modality (n (%))	515 (99%)	−0.33 (0.12–0.54)	0.00
Glasgow Coma Scale (n (%))	493 (95%)	−0.19 (−0.30–−0.08)	<0.001
Triage urgency (n (%))	517 (100%)	0.49 (0.12–0.86)	0.01
Age (n (%))	517 (100%)	0.02 (0.01–0.03)	0.00

Multivariate linear regression analysis of subjective and objective factors associated with clinical impression. Variables with a univariate association of *p* < 0.20 clinical impression score, age, gender, way of transport, triage, vital parameters, and Glasgow Coma Scale were selected for stepwise multivariate linear regression analysis (Supplementary Tables S2 and S3). Model characteristics: R^2^ = 0.42 (adjusted R^2^ = 0.40), df = 13, F = 18.65, *p* < 0.05. RC: regression coefficient, CI: confidence interval.

## Data Availability

Data used in this study can be obtained from Acutelines.

## Supplementary Materials

The following supporting information can be downloaded at: https://www.mdpi.com/article/10.3390/jcm13051359/s1, Figure S1: Case record form employed to obtain the clinical impression score and identify features associated with the clinical impression by physicians and nurses at the ED; Table S1: Results of the NTG workshop; Table S2: Univariate linear regression analysis of clinical impression features with the clinical impression score; Table S3: Univariate linear regression analysis of demographic factors and vital parameters with the clinical impression score; Table S4: Validation of multivariate linear regression model in infection group; Table S5: Validation of multivariate linear regression model in infection group.
