# Supplementary material for: Exploiting the Features of Clinical Judgment to Improve Assessment of Disease Severity in the Emergency Department: An Acutelines Study
Source: J Clin Med. 2024 Feb 27;13(5):1359. doi: 10.3390/jcm13051359 (PMC10931686; doi:10.3390/jcm13051359)
Supplement: Supplementary file 1 [file jcm-13-01359-s001.zip › jcm-2824724-supplementary.pdf]

Factors 1st round	Factors 2nd round
Work of breathing<br>Volume of speaking	*Breathing*<br>Frequency<br>Depth<br>Use of accessory muscles<br>Pace and volume of speaking<br>Breathing noises
Skin (color, mottled)<br>Feeling of the touch	*Skin*<br>Color<br>Aspect<br>Temperature
Awareness/consciousness	*Awareness/consciousness*
Eyes (color, shine, dullness)<br>Eye contact	*Eyes (glance)*
	Mucosae<br>Dehydration<br>Swelling<br>Blood
Age<br>Biological age	*Biological age-calandar age discrepancy*
Posture/attitude<br>Young patient not using smartphone<br>Eating or drinking	*Posture/attitude*
Observation of family members<br>Observation by general practioner (GP)<br>Stress ambulance/hectic environment<br>Observation nurse	*Behavior people around*
Mood (anxiety, agitation)<br>Mimicry/facial expression<br>Culture	*Frame of mind*
Smell	*Smell*
Selfcare<br>Nutritional status	*Self-care (neglect)*
Interventions ambulance	*Interventions ambulance*
Mobility after arrival at ED	*Mobility*
	Red flags physical examination
Presenting complaint<br>Feeling of faining	*Presenting complaints*
How ill does the patient feel<br>Patients feels that he/she is dying<br>Impression of family members	*Own impression*<br>Patient<br>GP<br>Family
Where does the patient come from (having a)<br>Network	*Socioeconomic status*<br>Living situation<br>Residence<br>Profession
Level of functioning	*Pre-existing level of functioning*
Transportation	*Type of transportation to hospital*
How late does the patient arrive	*Presenting at evening, night or weekends*

Medication<br>Medical history<br>Alcohol/smoking/drugs	*Patient information*<br>Medication<br>Medical history<br>Alcohol/smoking/drugs
Arterial blood gas analysis<br>Ultrasound	*Additional diagnostics*<br>ECG<br>ABG<br>Ultrasound
Impression of monitor	*Monitor vitals*
ABCDE	
Triage color	

Supplemental Table S1. Results of the NTG workshop

Clinical impression score

NB: for each item it is mandatory to provide an answer, where you would fill in "not applicable" fill in "no". Do not enter any identifying information on the form other than the participant number.

**Participant ID**		**Initials research assistant**	
**Date**<br>(DD-MM-JJJJ)	.... - .... - 20	**Signature Project Manager**	

**What is your position?**	O Student O Physician in training<br>O Medical specialist O Nurse		
**How many years of working experience have you had since graduating?**	.... year		
**How ill is the patient?**	[not ill] 1 - 2 - 3 - 4 - 5 - 6 - 7 - 8 - 9 - 10 [very ill]		
**Which items contributed significantly to your clinical impression?**			
Presenting complaint	yes / no	Behavior<br>Patient<br>Family<br>Ambulance staff<br>ED staff	 yes / no<br>yes / no<br>yes / no<br>yes / no
Way of transport to hospital	yes / no		
Comes in the evening, night or weekend	yes / no		
Ambulance interventions	yes / no		
Breathing<br>Frequency<br>Depth<br>Accessory muscles<br>Speaking-pace<br>Speaking-volume<br>Breath sounds	 yes / no<br>yes / no<br>yes / no<br>yes / no<br>yes / no<br>yes / no	Mood<br>Mimicry<br>Emotions	 yes / no<br>yes / no
		Mobility (e.g. from stretcher to bed, cooperation during physical examination, toilet use)	yes / no
Skin<br>Color<br>Aspect	 yes / no<br>yes / no	Estimation<br>of patient himself<br>of family	 yes / no<br>yes / no

Temperature	yes / no	of the GP	yes / no
Mucous membranes<br>Dry<br>Swelling<br>Blood	 yes / no<br>yes / no<br>yes / no	Worsening of functioning	yes / no
		Job	yes / no
		Living situation	yes / no
		Residence	yes / no
Smell	yes / no	Patient data<br>Medical history<br>Medication<br>Intoxication (alcohol, smoking, drugs)	 yes / no<br>yes / no<br>yes / no
Self-care / neglect	yes / no		
Consciousness	yes / no		
Eye glance	yes / no	Red Flags physical examination	yes / no
Calendar age	yes / no	Vitals bed-side monitor	yes / no
Estimation of biological age	yes / no	Additional diagnostics<br>ECG<br>Ultrasound<br>arterial blood gas analysis	 yes / no<br>yes / no<br>yes / no
Habitus (lying down, sitting, fetus)	yes / no		
Posture	yes / no		

Supplemental Figure S1. Case record form employed to obtain the clinical impression score and identify features associated with the clinical impression by physicians and nurses at the ED.

**Variables**	**% [n/N]**	**Regression Coefficient (95% CI)**	**p-value**
Presenting complaint	86% [436/510]	-0.07 (-0.59-0.46)	0.81
Way of transport to hospital	44% [222/513]	0.58 (0.22-0.95)	0.002
Comes in the evening, weekend, night hours	25% [127/508]	0.18 (-0.25-0.60)	0.42
Ambulance interventions	27% [136/508]	0.81 (0.39-1.22)	<0.001
Breathing			
Frequency	65% [337/517]	0.49 (0.11-0.87)	0.01
Depth	44% [227/515]	0.55 (0.18-0.91)	0.004
Use of accessory muscles	42% [213/513]	0.55 (0.18-0.92)	0.004
Speaking-pace	45% [231/513]	0.08 (-0.29-0.45)	0.68
Speaking-volume	37% [192/513]	0.35 (-0.03-0.73)	0.07
Breath sounds	40% [205/513]	0.49 (0.12-0.86)	0.01
Skin			
Color	51% [253/516]	0.75 (0.38-1.11)	<0.001
Aspect	30% [153/512]	0.58 (0.18-0.98)	0.005
Temperature	45% [233/512]	0.36 (-0.001-0.73)	0.05
Mucous membranes			
Dry	25% [126/515]	1.28 (0.87-1.69)	<0.001
Swelling	7% [36/513]	0.36 (-0.36-1.08)	0.33
Blood	9% [46/515]	0.57 (-0.07-1.21)	0.08
Smell	9% [48/510]	0.89 (0.26-1.52)	0.005
Selfcare/neglect	22% [113/513]	0.41 (-0.03-0.85)	0.07
Consciousness	55% [286/516]	0.15 (-0.22-0.52)	0.43
Eye glance	64% [328/516]	0.82 (0.44-1.19)	<0.001
Calendar age	28% [145/515]	0.46 (0.05-0.87)	0.03
Estimation of biological age	31% [156/512]	0.62 (0.22-1.01)	0.002
Habitus (lying down, sitting, fetus)	48% [245/513]	0.42 (0.05-0.78)	0.02

Posture	29% [148/514]	0.67 (0.26-1.07)	0.001
Behavior			
of the patient	61% [314/514]	-0.17 (-0.54-0.21)	0.39
of the family	18% [93/507]	-0.49 (-0.97- -0.02)	0.04
of the ambulance staff	19% [95/506]	0.63 (0.18-1.08)	0.01
of the ED staff	21% [104/506]	0.84 (0.37-1.31)	0.007
Mood			
Mimicry	37% [188/513]	0.33 (-0.05-0.71)	0.09
Emotions	29% [147/512]	-0.17 (-0.58-0.24)	0.41
Mobility	56% [283/510]	0.24 (-0.13-0.61)	0.21
Estimation			
of the patient himself/herself	35% [179/514]	-0.54 (-0.92- -0.16)	0.006
of the family	21% [106/510]	-0.21 (-0.66-0.25)	0.37
of the GP	22% [111/511]	0.57 (0.13-1.01)	0.01
Worsening in functioning	47% [241/515]	0.71 (0.35-1.07)	<0.001
Profession	2% [12/515]	0.79 (-1.14-1.29)	0.90
Living situation	14% [72/513]	0.96 (0.44-1.49)	<0.001
Residence	3% [16/511]	1.54 (0.49-2.60)	0.004
Medical history	78% [401/466]	-0.32 (-0.76-0.12)	0.16
Medication	43% [224/513]	0.20 (-0.17-0.57)	0.23
Intoxications (alcohol, nicotine, drugs)	16% [84/513]	0.14 (-0.36-0.64)	0.58
Red flags during physical examinations	57% [293/511]	1.26 (0.90-1.61)	<0.001
Vitals bed-side monitor	75% [381/510]	0.88 (0.46-1.30)	<0.001
Additional investigations			
ECG	34% [176/511]	0.11 (-0.28-0.50)	0.58
Ultrasound examination	16% [81/512]	1.04 (0.55-1.54)	<0.001
Arterial blood gas analysis	52% [269/514]	0.97 (0.61-1.32)	<0.001

Supplemental Table S2. Univariate linear regression analysis of clinical impression features with the clinical impression score. Shown is the relative and absolute number of cases where the health care professional identified the factor as contributing to the clinical impression score, as compared to the total number of respondents on the specific impression feature (% [n/N]). Mobility was judged on moving from stretcher tot bed, cooperation during physical examination, toilet use. RC: regression coefficient, CI: confidence interval.

**Variable**	**N (%) / median (IQR) / mean ±SD**	**RC (95% CI)**	**p-value**
Age	517 (100%)	0.03 (0.01-0.04)	<0.001
Gender	517 (100%)	-0.06 (-0.43-0.31)	0.74
Heart rate (bpm)	96 (69-123)	0.01 (0.003-0.02)	0.008
Systolic blood pressure (mmHg)	128 ± 23	-0.01 (-0.02- - 0.004)	0.004
Diastolic blood pressure (mmHg)	77 ± 17	-0.02 (-0.03- -0.01)	0.003
MAP (mmHg)	94 ± 18	-0.02 (-0.03- -0.01)	0.001
Respiration rate (rpm)	21 (13-29)	0.09 (0.06-0.12)	<0.001
Oxygen saturation (%)	96 (91-102)	-0.10 (-0.14- - 0.06)	<0.0001
Oxygen modality (%)	1 (0-2)	0.82 (0.63 – 1.00)	<0.001
Temperature (°C)	37.0 (35.8-38.2)	-0.08 (-0.23- -0.07)	0.28

Glasgow Coma Scale	15 (13-15)	-0.28 (-0.39- -0.18)	<0.001

Supplemental Table S3. Univariate linear regression analysis of demographic factors and vital parameters with the clinical impression score. IQR: interquartile range, RC: regression score, CI: confidence interval, bpm: beats per minute, MAP: mean arterial pressure, rpm: respirations per minute, SD standard deviation.

Variables	RC (95%CI)	p-value
CIS: Mucous membranes, dry	0.79 (0.28-1.29)	0.002
CIS: Eye glance	0.55 (0.07-1.03)	0.024
CIS: Behavior of the family	-0.97(-1.56- -0.37)	0.001
CIS: Estimation of the patient himself/herself	-0.74 (-1.20- -0.27)	0.002
CIS: Red flags during physical examinations	0.93 (0.47-1.38)	<0.001
Respiration rate (rpm)	0.57 (0.10-1.06)	0.018

Supplemental Table S4. Validation of multivariate linear regression model in infection group. Clinical impression features that associated with the clinical impression score in the multivariate regression model ($p<0.05$, Table 3) were validated in 307 patients with infection by associating the factors with the clinical impression score in a multivariate linear regression analysis (enter). RC: regression score, CI: confidence interval. Model characteristics: R^2=0.20, Adjusted R^2 =0.21, df=6 , F=11.68, $p<0.05$.

Variable	RC (95%CI)	p-value
CIS : Mucous membranes, dry	0.74 (0.21-1.28)	0.00
CIS: Eye glance	0.79 (0.31-1.28)	0.00
CIS : Estimation of the patient himself/herself	-0.67 (-1.16- -0.18)	0.01
CIS : Red flags during physical examinations	1.01 (0.56-1.46)	.<0.001
CIS: Behavior family	-0.80 (-1.40- -0.19)	0.01
Triage urgency	0.78 (0.31-1.26)	0.00
Heart rate (bpm)	0.02 (0.01-0.03)	<0.001
Respiration rate (rpm)	0.04 (-0.002-0.07)	0.03
Oxygen modality	0.25 (-0.006 – 0.51)	0.06
Age	0.02 (0.004-0.04)	0.02
Temperature	-0.19 (-0.39- -0.002)	0.05

Supplemental Table S5. (Table S5 Validation of multivariate linear regression model in infection group. Clinical impression features, vital parameters and demographic data that associated with the clinical impression score in the multivariate regression model ($p<0.05$, Table 4) were validated in 307

patients with infection by associating the factors with the clinical impression score in a multivariate linear regression analysis (enter). RC: regression score, CI: confidence interval. Model characteristics: R^2=0.43, Adjusted R^2=0.40, df=13, F=14.82, $p<0.05$.
